# Establishment of planar cell polarity is coupled to regional cell cycle exit and cell differentiation in the mouse utricle

**DOI:** 10.1038/srep43021

**Published:** 2017-02-23

**Authors:** Xiaoyu Yang, Xiaoqing Qian, Rui Ma, Xinwei Wang, Juanmei Yang, Wenwei Luo, Ping Chen, Fanglu Chi, Dongdong Ren

**Affiliations:** 1 Department of Otology and Skull Base Surgery, Eye & ENT Hospital of Fudan University, Shanghai 200031, China; 2 Shanghai Clinical Medical Center of Hearing Medicine, Eye & ENT Hospital of Fudan University, Shanghai 200031, China; 3 Department of Research Center, Eye & ENT Hospital of Fudan University, Shanghai 200031, China; 4 Department of Cell Biology, Emory University, Atlanta, GA 30322, USA

## Abstract

Sensory hair cells are coordinately oriented within each inner ear sensory organ to exhibit a particular form of planar cell polarity (PCP) necessary for mechanotransduction. However, the developmental events associated with establishing PCP in the vestibule are unclear, hindering data interpretation and employment of the vestibule for PCP studies. Herein, we investigated PCP of the mouse vestibular organs. We further characterised cell cycle exit, cell differentiation, and PCP establishment in the utricle. We found that hair cells formed first in the striolar and medial extrastriolar (MES) regions of the utricle at embryonic day 11.5 (E11.5), while cells in the lateral extrastriolar region (LES) mostly formed at E13.5. Cell differentiation was initiated in the striolar region, which expanded first toward the MES, then to the LES by E15.5. The polarity of hair cells was established at birth along a putative line of polarity reversal (LPR), lateral to the striolar region. Core PCP protein Vangl2 emerged in the cell boundaries since E11.5, while cell intrinsic polarity protein Gαi3 appeared at E12.5, then polarized to the bare zone of individual hair cell at E13.5. These findings provide a blueprint of the developmental events associated with establishing PCP in the utricle.

The mammalian inner ear is composed of six sensory organs with differing functions: the organ of Corti in the cochlea detects airborne sound vibrations; the maculae contained within the utricle and saccule in the vestibule detect linear acceleration; and three cristae at the ends of semicircular canals in the vestibule detect angular acceleration^1^. The detection and transformation of mechanical signals to their corresponding neural pathways depend on the integrity and polarity of the stereocilia bundles that adorn the apical surface of each sensory hair cell^2^,^3^. Abnormalities in the organisation and polarity of stereocilia bundles result in balance and hearing defects in humans and mice^4^,^5^,^6^,^7^,^8^. Moreover, in the inner ear, the hair cells of sensory organs are coordinately oriented, displaying unique forms of planar cell polarity (PCP)^9^. The coordinated orientation of hair cells in each sensory organ is vital for their individual functions in hearing and balance^10^. The relative orientation of hair cells in all five vestibular sensory organs is essential for balance in three-dimensional (3D) space^11^. The sensory organ of the saccule or the utricle, comprising a sheet of sensory hair cells interdigitated with non-sensory supporting cells, is known as the macula. The relative orientation of the hair cells on the macula is essential for sensing linear acceleration and head tilt. The sensory organs at the ends of the semicircular canals, the crista ampullae, are dumbbell-shaped, and the perpendicular orientation of the three cristae is responsible for sensing head rotation or angular acceleration in 3D space^1^,^11^.

PCP is achieved by coordinated orientation of intrinsically polarised cells within a tissue. In vertebrates, PCP is regulated by vertebrate-specific PCP genes and a set of core PCP genes that are conserved across species, from *Drosophila* to humans^1^,^11^. The conserved core PCP genes include *Dishevelled (Dvl*), *Frizzled (Fzd*), *Prickle (Pk*), and *Van Gogh-like (Vangl*) genes. These genes encode membrane core PCP proteins and their associated proteins to form opposing PCP complexes at cellular junctions, which are formed between adjacent cells to coordinate the polarity among neighbouring cells using cell-autonomous and non-cell-autonomous mechanisms^12^,^13^. In addition, vertebrate-specific PCP genes, such as the basal body and cilia genes, regulate the intrinsic polarity of cells in a cell-autonomous manner^14^,^15^. G protein Gαi plays an important role in emergence of asymmetric stereocilia bundles of hair cell. Insc- Gαi -LGN complex is located in the lateral microvilli-free region, or “bare zone” at the apical surface, while aPKC-Par3-Par6 expression is opposite and complementary^16^,^17^. Gαi3 and LGN also occupy the very tip of stereoclia that directly abut the bare zone^18^. Defects in core PCP or cilia/basal body genes lead to the misalignment of neighbouring cells or loss of the intrinsic polarity of cells, respectively^19^,^20^.

The establishment of PCP during development is uniquely coupled to other development events in each tissue. In the cochlea, the developmental events associated with terminal differentiation when PCP is established have been clearly delineated, facilitating the application of the cochlea as a model system for vertebrate PCP studies^12^,^21^. Subsequent to terminal mitosis, differentiation of the organ of Corti starts closely to the base of the cochlea and extends toward the apex^22^,^23^,^24^,^25^. Once hair cells differentiation is complete, the stereociliary bundles are organised. Initially, a single primary cilium emerges at the centre of the apical surface and is surrounded by microvilli of uniform height. Afterwards, the primary cilium, known as the kinocilium for sensory hair cells, begins to migrate toward the lateral side of the apical surface and is positioned asymmetrically. In the cochlea, this process results in the formation of a staircased and V-shaped stereocilia bundle with the tallest stereocilia near the kinocilium. In the cochlea, the PCP manifests in another form, called convergent extension (CE). The uniform orientation of sensory hair cells in the organ of Corti occurs concomitantly with cellular boundary remodelling that accompanies cellular rearrangements during CE^9^,^21^,^26^.

In the vestibule, PCP is represented at three distinct anatomical scales within the maculae of the utricle or the saccule^27^. First, at the intercellular scale, the molecular basis of planar polarity is determined by core PCP proteins such as Dvl2/3, Fzd3, Pk, and Vangl2 that coordinate the orientation of stereociliary bundle polarity among neighbouring cells. Second, each hair cell has a precisely oriented stereociliary bundle on the apical cell surface^28^. At embryonic day 12.5 (E12.5) in mice, the first hair cells can be distinguished from precursors by the specialisation on their apical surface. A single primary cilium is present from the centre of the apical surface and is surrounded by elongated microvilli, which later become stereocilia. This primary cilium elongates to form the kinocilium and appears to be relocated to one side of the apical surface of the hair cells, providing the first evidence of intrinsic cellular polarity of individual hair cells^28^. Third, cellular polarity in the vestibular maculae varies within the organ. Utricular hair cells are oriented with their bundles pointed toward each other along a putative line of polarity reversal (LPR), while hair cells in the saccule of the stereocilia bundles point away from each other along the LPR^28^,^29^. Significantly, core PCP proteins are located in the same asymmetric manner independent of the intrinsic polarity of hair cells, implicating additional mechanisms in determining the polarity of individual hair cells^30^,^31^. Despite the unique features of PCP in the vestibular maculae that might offer a unique opportunity to address critical issues in vertebrate PCP regulation, the vestibular organs have not been used extensively for PCP studies. The application of the vestibule for PCP studies and data interpretation is hindered by the lack of understanding regarding the key developmental events during the establishment of PCP, the imprecise characterisation of PCP relative to the major anatomic landmarks of the vestibular maculae, and the difficulty in preparing intact sensory epithelia for PCP investigation.

Here, we present a comprehensive description of PCP in the five sensory organs of the mouse vestibular system at E18.5. We selected the utricle for the systematic investigation of critical developmental events, including terminal cell division and cell differentiation within specific regions of the utricle, relative to the establishment of PCP and the LPR. In addition, we detected the expression of cell-intrinsic polarity protein and core PCP protein in early development stage. The cell intrinsic polarity protein localization provided a comprehensive report of the establishment of various aspects of PCP in the developing utricle. This study provides guidelines for referencing PCP within different regions of the utricle, suggests the presence of distinct regional cues for regulating hair cell polarity across the LPR. The information presented in the current study is essential for data interpretation of developmental regulations, including PCP regulations, in the utricle.

## Results

### Relative PCP of vestibular sensory organs in mice

The vestibule comprises five sensory organs, each with sensory epithelial sheets in the maculae of the utricle and saccule, and in the three semicircular canals with dumbbell-shaped cristae at the ends. Hair cells in the maculae are of opposing polarity, whereas hair cells in the cristae are oriented uniformly. However, the organisation of hair cells based on their polarity and physiological function in each of the vestibular organs and their relationship with the other sensory organs is unclear.

The utricle and saccule can be divided into three regions: the central striola, the peripheral extrastriola composed of the medial extrastriola (MES), and the lateral extrastriola (LES). There are two types of hair cells (type I and type II) in the maculae and cristae. Type I hair cells, located at the centre of the maculae and at the top of the cristae, are the first cells in their corresponding organ to be innervated^32^.

Type I hair cells have a wide array of stereocilia, with the kinocilium near the tallest stereocilia. They are large and fast conduits for phasic signals encoded in irregular spike trains. Type II hair cells have a long kinocilium that extends deeply into the overlying layers, and they have a very narrow array of short and fine stereocilia. The central type I hair cells of vestibular organs conduct signals to large afferents with phasic (adapting) response dynamics and high conduction speeds, while peripheral afferents to type II hair cells have complementary properties^33^. The finer afferents from peripheral zones of the maculae transmit more slowly and encode more tonic, linear signals in highly regular spike trains. Both maculae consist of a crescent-shaped region known as the striola where the type I hair cells are located^34^.

**Figure 1:**
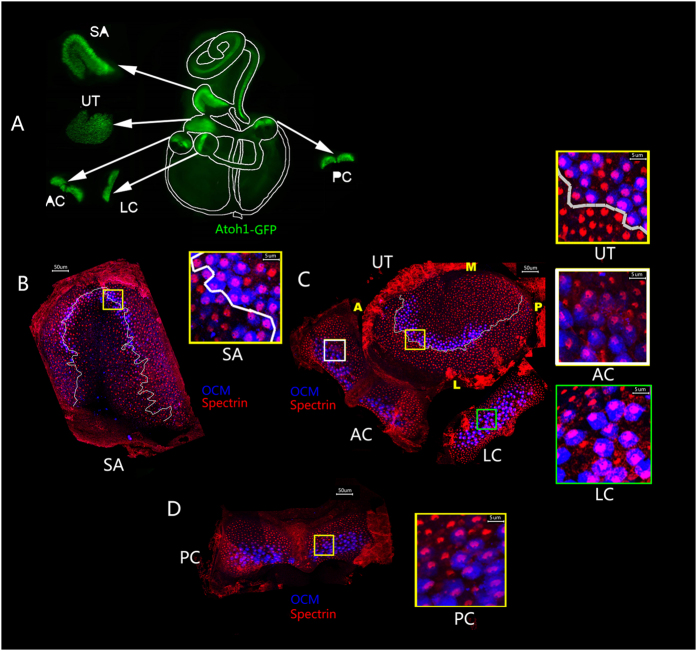
Planar cell polarity (PCP) in the mouse vestibule. (**A**) An overview of the mouse vestibular system. (**B**) PCP of the saccule (SA). The line of polarity reversal (LPR, white line) is located within the striola, marked by the OCM^+^ (blue) type I hair cells. Hair cells are oriented with their fonticulus visible and stereociliary bundles pointed away from the LPR. (**C**) PCP of the utricle (UT), anterior cristae (AC), and lateral cristae (LC). In the utricle, the LPR is located lateral to the striola. Hair cells are oriented with their fonticulus visible and stereociliary bundles pointed toward the LPR. Hair cells in the lateral cristae are oriented in a manner similar to the hair cells lateral of the LPR in the utricle. Hair cells in the anterior cristae are oriented in a manner similar to hair cells medial of the LPR in the utricle. (**D**) PCP of the posterior cristae (PC). The hair cells are oriented posteriorly in the same direction as those medial to the LRP in the utricle. The boxed regions in (**B–D**) were all presented at a higher magnification. Atoh1/EGFP (green) marks all hair cells; β-spectrin (red) labels the actin-rich cuticular plate; OCM (blue) is expressed in type I hair cells in the striola. Scale bar: 50 μm.

The polarity of a single hair cell is reflected by the orientation and patterning of the stereocilia bundle and the positioning of the kinocilium. The fonticulus is devoid of β-spectrin staining, and marks the location where the basal body is located, from which the kinocilium is projected near the tallest stereocilia. Therefore, the location of the fonticulus is indicative of the orientation of hair cells. We used *Atoh1/EGFP* reporter mouse to mark the sensory epithelium^35^, β-spectrin to visualise the fonticulus of the cuticular plate, and oncomodulin (OCM) to label type I hair cells^36^ in the maculae (Fig. 1). The combination of β-spectrin and OCM staining with Atoh1/EGFP visualisation allowed us to locate the relative position of the striola in the maculae on a gross level.

In the saccule, OCM^+^ type I hair cells in the striolar region were oriented with their fonticulus pointing away or toward the periphery of the saccule to create a putative LPR within the striola (Fig. 1B). Hair cells on either side of the LPR were oriented uniformly (Fig. 1B). In the utricle, hair cells in the striolar region, where OCM^+^ type I hair cells were located, and in the region medial to the striola or MES, were oriented toward the periphery of the utricle (Fig. 1C). In contrast, hair cells in the region lateral to the striola or LES were oriented toward the centre or medial side of the utricle. The opposing orientation of hair cells in the LES and in the striolar and MES regions created a notional LPR that outlined the lateral edge of the striola in the utricle (Fig. 1C). In the lateral cristae, hair cells were oriented similarly to those located in the LES of the utricle, whereas hair cells in the anterior cristae were oriented in the same direction as those in the striola and MES of the utricle (Fig. 1D). Hair cells in the posterior cristae were oriented posteriorly (Fig. 1D).

### Regionally specific terminal cell divisions and expansion of the postmitotic developing sensory epithelium marked by cyclin-dependent kinase inhibitor p27^Kip1^ in the utricle

The utricle and saccule are composed of hair cells of reverse orientation, providing a unique opportunity to examine the mechanisms of directional cues for PCP regulation across the tissue. It is often difficult to obtain intact flattened saccule whole mounts, as the saccule has a greater curvature. We selected the utricle to characterise the key developmental events during terminal differentiation and establishment of PCP in the vestibular organs.

**Figure 2:**
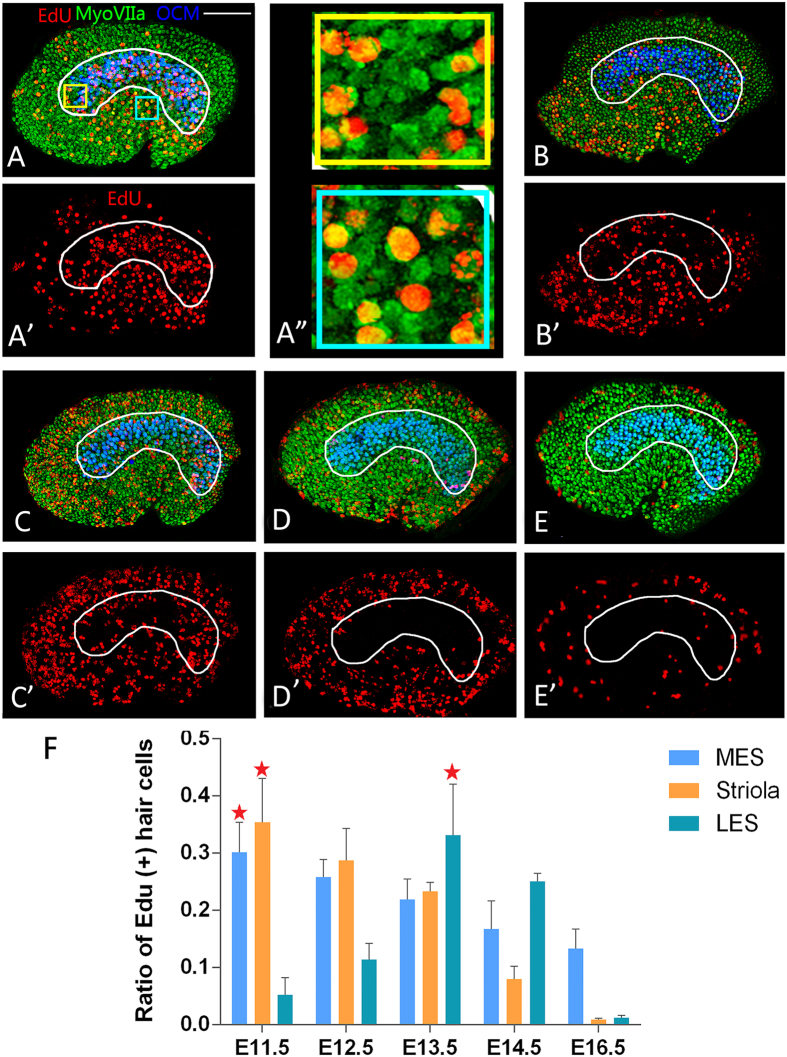
Regionally specific terminal cell divisions in the developing utricle. Pregnant female mice were injected once with 5-ethynyl-2′-deoxyuridine (EdU) to label cells in S phase at embryonic day (**E**)11.5 (**A**, A’, A”), E12.5 (**B**, B’), E13.5 (**C**, C’), E14.5 (**D**, D’), and E16.5 (**E**, E’), and allowed to survive until E18.5. Each sample was stained for EdU to mark the cells in the S phase of the cell cycle at the time of injection, and with an antibody to myosin VIIa for hair cells and OCM for type I hair cells in the striola. Scale bar: 100 μm. Fields in yellow and blue box (A”) were chosen from striola and MES respectively for a higher magnification image. We only counted double labelled cells for statistical analysis. The percentages of hair cells born at each stage in each of the three segments of the utricle, striola, medial extrastriola (MES), and lateral extrastriola (LES) (**F**). At E13.5, the ratios of EdU and myosin VIIa double-positive hair cells to the total number of hair cells were 0.331 ± 0.089, 0.233 ± 0.016, and 0.219 ± 0.036 in LES, striola, and MES, respectively (P* < *0.01, n = 5). At E16.5, the ratios of EdU and myosin VIIa double-positive hair cells to the total number of hair cells were 0.133 ± 0.034, 0.009 ± 0.002, and 0.012 ± 0.004 in LES, striola, and MES, respectively (P* < *0.01, n = 5).

To determine the peak of terminal cell divisions in the utricle, we pulse-labelled cells at the S phase of the cell cycle with the nucleotide analogue 5-ethynyl-2′-deoxyuridine (EdU) at different developmental stages, and harvested the tissues at the end of embryonic development. We also used myosin VIIa to label hair cells and marked the striola with OCM (Fig. 2A–E’). The ratio of EdU^+^ and myosin VIIa^+^ cells to the total number of myosin VIIa^+^ hair cells was used to quantify proliferation in each region at each stage (Fig. 2F). We only counted cells that both Edu and myosin VIIa were positive (Fig. 1A”). In the striolar region of the utricle, proliferation peaked at E11.5, significantly decreased at E12.5, and was nearly absent by E14.5. Similarly, cell proliferation was already underway by E11.5 in the MES, and continued to decrease until E16.5 when cell proliferation was scarcely detected. In the LES, proliferation levels were low at E11.5, peaked at E13.5 and decreased significantly from E13.5 to E16.5. These findings indicate that the precursors of hair cells in the striola and MES begin terminal mitosis before those in the LES of the utricle.

**Figure 3:**
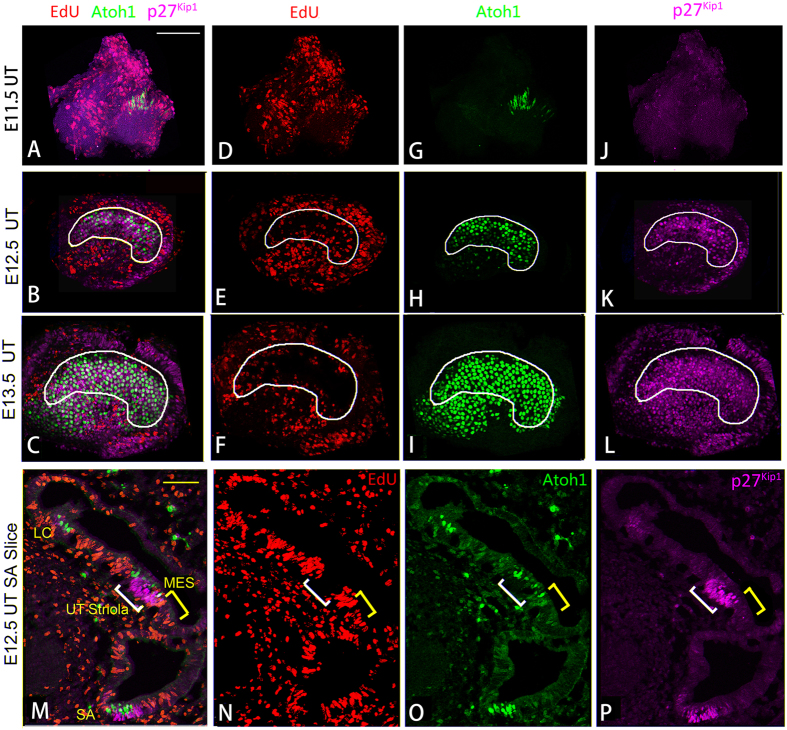
Cyclin-dependent kinase inhibitor p27^Kip1^ expression demarcates the expansion of the post-mitotic sensory domain within the developing utricle. Pregnant *Atoh1/EGFP* female mice were injected with EdU three times at 2-h intervals at E11.5 (**A,D,G,J**), E12.5 (**B,E,H,K,M–P**) or E13.5 (**C,F,I,L**), and utricle samples were harvested 6 h after the initial injections. Whole-mount (**A–L**) and cross-section (**M–P**) preparations of harvested utricles were stained for EdU and p27^Kip1^. No p27^Kip1^ expression was found at E11.5, while Atoh1/EGFP expression occurs early (**A,D,G,J**). The expression of P27^Kip1^ marked the same domain of minimum EdU incorporation where the early formations of Atoh1/EGFP^+^ hair cells were located at E12.5 (**B,E,H,K**). The p27^Kip1^ domain expanded, overlapping with the increased Atoh1/EGFP^+^ sensory domain, which was devoid of EdU incorporation at E13.5 (**C,F,I,L**). It appeared that the developing sensory domain expanded from the striolar region (outlined by white lines) toward the MES from E12.5 to E13.5 (**B–L**). Cross sections (**M–P**) revealed the developing sensory epithelia in the utricle, saccule and the lateral crista(LC). White and yellow brackets mark the striolar and MES regions of the utricle, respectively. Scale: 100 μm.

To assess and confirm the region-specific terminal mitosis in the utricle, we performed a shorter EdU chase in combination with hair cell and cell cycle markers. We injected EdU into pregnant *Atoh1/EGFP* reporter mouse at E11.5, 12.5 or E13.5, and stained for EdU and cyclin-dependent kinase inhibitor p27^Kip1^. p27^Kip1^ marks the postmitotic prosensory domain that becomes sensory hair cells and non-sensory supporting cells in the cochlea and in the supporting cells subsequent to hair cell differentiation^22^,^37^. At E11.5, No p27^Kip1^ expression could been seen, while Atoh1/EGFP expression occurs early in the middle of the utricle (Fig. 3A,D,G,J). At E12.5, p27^Kip1^ and Atoh1/EGFP marked the same central region corresponding to the striolar region of the utricle that had less incorporation of EdU than the MES and LES regions (Fig. 3B,E,H,K,M–P). At E13.5, the region marked by p27^Kip1^ and Atoh1/EGFP that was devoid of EdU incorporation expanded toward the MES (Fig. 3C,F,I,L).

The short and long EdU chase experiments confirmed that proliferation of hair cell precusors peaked at E11.5 for cells in the striola, and most of cells proliferation extended in the MES and LES until E13.5–E14.5 and E16.5, respectively. The data also revealed that p27^Kip1^ marks the postmitotic and terminally differentiating sensory region in the developing utricle.

### Planar cell polarity establishment in the utricle

**Figure 4:**
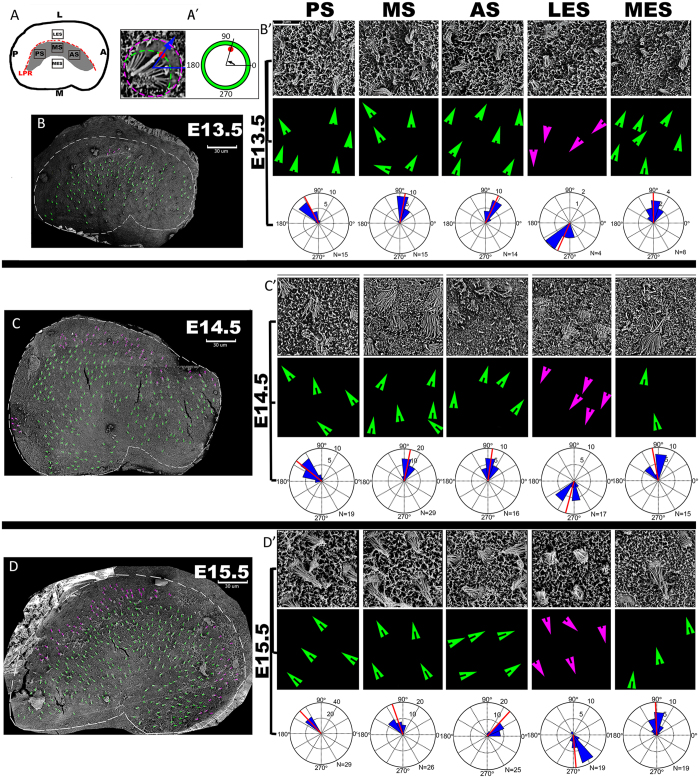
The polarity of hair cells is established at their birth along the LPR in the utricle. E13.5 (**B**,B’), E14.5 (**C,**C’), and E15.5 (**D**,D’) mouse utricles were imaged using SEM (8000 × ) and images were combined to generate the overall views. Arrows mark the orientation of individual developing hair cells based on the location of their kinocilium. The method of measurement of hair cell orientations was showed (A’). Five fields chosen from the whole epithelium (**A**) were presented at a higher magnification (in B’,C’, and D’, respectively). The locations of kinocilia were marked by magenta or green dots for hair cells lateral or medial to the putative LPR, respectively. At E13.5, hair cells were visible in the striolar and MES regions, and stereociliary bundles of hair cells in the striolar region were oriented mostly toward the LPR (**B**,B’). At E14.5, hair cells started to appear in the LES region, and the polarity of hair cells on either side of the LPR was established (**C**,C’). By E15.5, the polarity of hair cells was distinctively visible across the utricle in the striolar, MES, and LES regions (**D**,D’). Scale: 30 μm. Circular histograms demonstrating the orientations of all of the vestibular hair cells that were measured from fields1–5 for different embryo stages (B’,C’,D’). The number of cells in each bin is graphed along the x-axis and the cell total is listed. The average orientation of each group of cells is marked by a red line.

Using scanning electron microscopy (SEM), we investigated the timing of the establishment of PCP in the utricle (Fig. 4). At E13.5, few hair cells were present in the lateral region, while more hair cells were present in the medial region of the utricle (Fig. 4B,B’). However, the few hair cells in the lateral region had already adopted a direction that opposed the direction of hair cells in the medial region, across an imaginary LPR. Considering the data for terminal mitosis and differentiation (Figs 2 and 3), the lateral region consisting of fewer hair cells is the LES, and the medial region with a greater number of hair cells includes both the striola and the MES. These findings suggest that the regional specification in the utricle precedes the terminal differentiation of hair cells, and imply that hair cells adopt their terminal orientation as soon as they are differentiated. Consistently, hair cell numbers had increased significantly at E14.5 in the LES lateral to the LPR that had been clearly established, and they were oriented in the medial direction, opposing the orientation of hair cells in the striola and the MES (Fig. 4C,C’). This trend of hair cell differentiation continued at E15.5 (Fig. 4D,D’). Along with the growth of age, we could found that both the number and the extent of organization of hair cells increased. These trends were quantified by graphing the orientation (from 0–360°) of individual hair cells from all embryos using circular histograms(Fig. 4B’,C’,D’).

**Figure 5:**
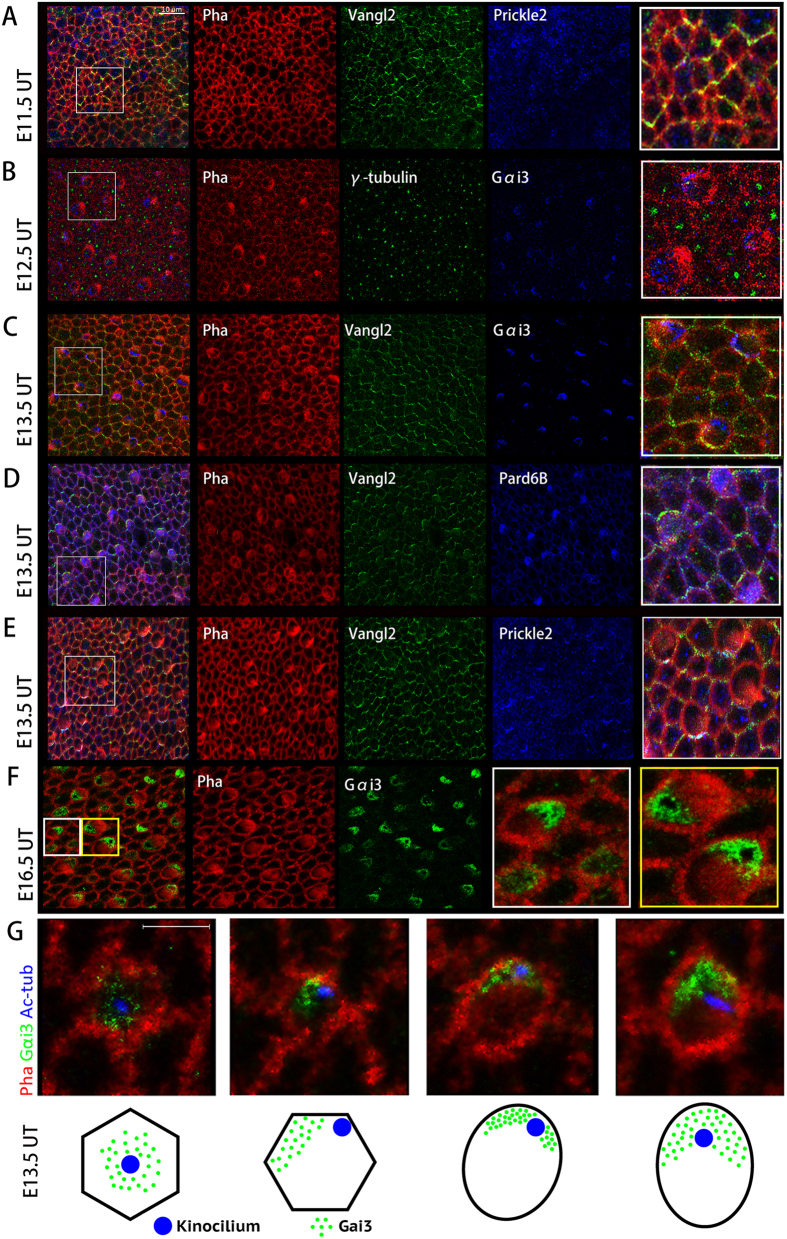
The cell-intrinsic polarity proteins and core PCP protein expression in the utricle. E11.5 (**A**), E12.5 (**B**), E13.5 (**C–E,G**) and E16.5 (**F**) mouse utricles were imaged. At E11.5, Vangl2 was expressed in the cell boundaries of utricle before hair cell polarity formation, while prickle2 didn’t emerge (A). At 12.5, cell-intrinsic protein Gαi3 started to appear in the utricle (**B**). At E13.5 when the LPR began to form, Gαi3 was found in the bare zone of hair cell (**C**), Pard6B was on the opposite side (**D**), Vangl2 and Prickle2 located at the same side of hair cell though they were two groups of hair cells positioned at the different sides of the LPR (**E**). At E16.5, different hair cells were not in the same development stages (**F**). In early development stage, Gαi3 was located in the whole cytoplasm of hair cell (White box). In later stage, Gαi3 moved to the bare zone (Yellow box). Schematic representation of hair cells in different development stage in E13.5 utricular epithelium (**G**). Gαi3 was gradually polarized to the bare zone, and kinocilium relocalized from centre to one side of hair cell. Fields in white or yellow box were chosen for a higher magnification image. Scale bars: 10 μm (**A–F**), 5 μm (**G**).

To explore the mechanism of how tissue polarity was established, we also examined the expression of cell-intrinsic polarity proteins Gαi3 and Pard6B, and core PCP protein Vangl2 and Prickle2 in the embryo utricle. At E11.5, Vangl2 was expressed in the cell boundaries of utricle before hair cell polarity formation, while prickle2 (Fig. 5A) and Gαi3 (data not shown) didn’t emerge. At 12.5, Gαi3 started to appear in the utricle (Fig. 5B), while Prickle2 still could not be found (data not shown). At E13.5, when the LPR began to form, Gαi3 was found in the bare zone of individual hair cell (Fig. 5C), Pard6B was on the opposite side (Fig. 5D), they were located on the opposite side of two different groups of hair cells positioned at the different sides of the LPR, while core PCP protein Vangl2 and Prickle2 located at the same side of hair cells (Fig. 5E). At both E13.5 (Fig. 5G) and E16.5 (Fig. 5F), we could see that different hair cells were not in the same development stages. In early development stage, Gαi3 was located in the whole cytoplasm of hair cell, then for cells in the later development status, Gαi3 moved to the bare zone (Fig. 5G). The experiment confirmed that core PCP protein established earlier and later individual polarity signalling was activated within differentiating hair cells. Tissue polarity was subsequently established after the orientation of every single hair cell of the tissue was coordinated.

## Discussion

The polarity of individual hair cells, the coordinated orientation of hair cells within each sensory organ, and their effect on other sensory organs in the vestibule are essential for directional responses to head movements. Hair cells of opposing directions within otolithic maculae are separated into two domains; therefore, membrane hyperpolarisation in one group of macular hair cells and depolarisation in the other group will be evoked by head tilt or an arbitrary linear acceleration. In contrast, in the cristae, hair cells of the semicircular canals of a sensory end organ are orientated uniformly and display similar responses to a given rotational stimulus^33^. Indeed, we showed that hair cells in the maculae of the utricle and saccule are composed of hair cells with opposite orientations, whereas those in the cristae are uniformly oriented (Fig. 1).

This study revealed new findings regarding the organisation of the maculae. In the maculae of the utricle and saccule, hair cells with opposing orientations are distributed along an imagined LPR^28^,^38^. The precise position of the LPR, however, is vague. Both maculae consist of a crescent-shaped region known as the striola where the type I hair cells are located. We used OCM, a molecular marker that specifically labels the calyces of type I hair cells^36^, to reference the striola and determined the LPR relative to the striola. We found that the LPR in the utricle is lateral to the striolar region, consistent with a report by Li *et al*.^39^, and the type I hair cells are in the same direction in the striola of the utricle (Fig. 1). The LPR maintains the trajectory of the striola anteriorly; however, it departs from this trajectory posteriorly, running toward the posterior margin of the maculae^39^ In contrast, in the saccule, the LPR is in the middle of the striola, and the type I hair cells in the striola are oriented in two opposing directions in the striola of the saccule (Fig. 1). The specification of the striola relative to the LPR may have two implications for signal processing from the left and right labyrinths. First, only certain head movement directions would trigger monosynaptic striolar drive to secondary neurons in mice. Second, the LPR may be related to the generation of the translational vestibulo-ocular reflex (TVOR)^39^. The differing positions of the LPR in the utricle and saccule imply that the developmental regulation and/or the mechanism underlying the reverse polarity of hair cells in the two maculae may differ.

In addition to characterising hair cell polarity within each sensory organ, we provided the first comprehensive schematic view of PCP in the five sensory organs in the vestibule relative to each other (Fig. 1A). Hair cells in the lateral crista are oriented in a manner similar to the LES of the utricle. Hair cells in the anterior semicircular canal were positioned in an orientation similar to the hair cells in the striola and MES of the utricle (Fig. 1C). In the posterior crista, hair cells were oriented posteriorly (Fig. 1D). The relative positions of type I hair cells in the crista were also determined (Fig. 1). The overall view of PCP and organisation of hair cell types in the vestibule is useful for interpreting the physiological functions of the vestibule and the pathogenesis of the system.

The distinct and opposing polarities of hair cells in the macula of the utricle or the saccule offer a rare opportunity to address fundamental issues in PCP regulation, such as the nature of the global directional cues and how these cues act with core PCP and cilia/basal body genes to coordinate the polarity of cells across the tissue. However, developmental studies of the vestibule are scarce, and the key events that would aid data interpretation and application of the vestibular maculae for PCP studies have not been well documented. In this study, we characterised and completed the groundwork to establish the time course of cell proliferation, cell cycle exit, cell differentiation, and the formation of PCP in the utricle during development.

In the cochlea, the precursor cells that give rise to hair cells and supporting cells exit the cell cycle and undergo terminal differentiation in reverse gradients along the longitudinal axis of the cochlea^25^. In contrast, cell cycle exit and terminal differentiation in the utricle follow the same expansion pattern, from the striola and MES to the LES, consistent with a previous study that revealed that hair cells differentiate in a roughly central-to-peripheral direction from E13.5 to the first postnatal week^40^,^41^. Moreover, previous studies showed that a cyclin-dependent kinase inhibitor, p27^Kip1^, is expressed in the differentiating vestibule as early as E14 in the supporting cells^25^. In this study, we detected the onset of p27^Kip1^ in the utricle as early as E12.5, corresponding to the earliest born precursor cells in the striola of the utricle. The expression domain of p27^Kip1^ extends to the MES and LES, similar to the increase in Atoh1/EGFP and decrease in EdU incorporation in the utricle. Similar to the time course observed in the cochlea, it is likely that p27^Kip1^ marks the postmitotic precursors that give rise to both hair cells and supporting cells, and is subsequently restricted to the supporting cells in the utricle.

To establish the earliest time when the polarity of hair cells can be observed, we used scanning electron microscopy (SEM). As early as E13.5, morphologically recognisable hair cells were polarised mostly toward the periphery of the utricle, while a few hair cells with opposite polarity were localised lateral to those pointing outwardly. As development progressed to E14.5 and E15.5, we observed an increased number of hair cells in the lateral area that pointed medially. These findings were informative regarding the specification of the two domains of hair cells with opposite orientations, the LES versus the striola and the MES, and the existence of polarity cues for hair cells in both domains by E13.5. The high resolution of SEM revealed the earliest known stage for hair cell polarity across the LPR in the utricle^38^. Interestingly, regenerated hair cells are correctly oriented following gentamicin-induced hair cell loss in the utricles of adult guinea pigs^42^ or streptomycin-induced damage in the avian utricle^43^. It is possible that similar directional cues, as well as additional instructions from core PCP protein complexes in the neighbouring cells, may direct the orientation of regenerating hair cells.

Afterwards we performed experiments to test the expression of cell-intrinsic polarity protein Gαi3 and Pard6B, core PCP protein Vangl2 and Prickle 2. We found that Vangl2 emerged in cell boundaries of utricle since E11.5, later Gαi3 began to express at E12.5. When the LPR was formed at E13.5, Prickle2 appeared and Gαi3 was polarized to the bare zone of an individual hair cell. We also observed that Gαi3 positioned from the whole epithelium to the bare zone in hair cells of different development status at E13.5. Moreover, Gαi3 and Pard6B were located reversely in different side of LPR, while core PCP protein were at the same side of utricular hair cells. In cochlea, when the intercellular PCP signal mediated by core PCP proteins is interrupted, the Insc/Gαi/LGN complex still localizes on the apical surface of hair cells asymmetrically, but no longer along the medial-lateral direction, as observed in core PCP mutant mice^16^,^17^. When Insc/Gαi/LGN complex was interrupted, the autonomous hair cell PCP was disturbed leading to the misorientation of hair cells; however, the asymmetric localizations of core PCP proteins remain unaffected^16^,^17^. We draw a conclusion that core PCP protein signalling established earlier in the utricle hair cells and then gives cues to cell-intrinsic polarity protein to instruct the migration of kinocilium. The two signalling pathway work together to coordinate orientation throughout the whole epithelium. Vangl2 may play a key role in the polarity establishment. But the mechanism of how the signal is transduced to regulate PCP is still unknown. Sun *et al*. hypothesized that GPCR- Gαi coupling, for example Celsr and Frizzled in the upstream signaling, played a pivotal role in cochlear PCP regulation via connecting the intercellular PCP signals with cell-autonomous PCP machinery in cochlea^44^. Nevertheless, how could the same cell-intrinsic polarity protein instruct opposite cell polarity under the direction of the same core PCP signals remains intricate.

In summary, precursor cells of the utricle macula exit the cell cycle and undergo terminal differentiation in the same spatial order in a central-to-peripheral progression, beginning in the striola followed by cells in the MES, then the LES. Moreover, the specification of the LES versus the striola and the MES, as well as the directional cues for cells across the LPR, precedes the differentiation of hair cells in the LES.

The early specification of the two domains across the future LPR in the utricle suggested that the two domains may have different molecular identities and/or specific molecular cues at the boundary of the two domains to direct the orientation of hair cells on either side of the LPR.

In mice, targeted or spontaneous mutations in core PCP genes, such as Celsr1, Vangl2, or Fzd3 and Fzd6, lead to randomisation of hair cells in the utricle^45^,^46^,^47^,^48^. The core PCP proteins encoded by these conserved PCP genes show asymmetric and polarised localisation at the boundaries formed between neighbouring cells across the utricle at the stage when PCP has been established^9^. Studies in the localisation and the loss-of-function of these core PCP proteins suggested that they act to coordinate cell polarity, rather than to drive specification of the two domains across the LPR for providing the directional cues to the hair cells^29^.

The mechanisms underlying the specification of the two domains with opposite hair cell direction and the directional cues in the utricle remain unknown. A blueprint of cell cycle exit, terminal differentiation, and PCP formation in the utricle provides an invaluable reference when applying this unique model system to addressing remaining questions in PCP regulation in vertebrates.

## Methods

### Animal protocols

All experimental procedures, performed strictly under the guidelines of the Ethical Board of Eye & ENT Hospital of Fudan University, were approved by the Chinese Science Academy Committee on Care and Use of Animals. Wild-type (WT) C57BL/6 mice were purchased from Shanghai SLAC Laboratory Animal Co., Ltd. (Shanghai, China). *Atoh1/EGFP* reporter C57BL/6-mice were purchased from the Jackson Laboratory (Bar Harbor, ME, USA) (*Atoh1*^*tm4.1Hzo*^, stock no. 013593). The *Atoh1/EGFP* transgene contains an ~1.4 kb sequence from the *Atoh1* enhancer (Tg15 plus 150 bp on the 3′end) fused to the reporter gene BGnEGFP. Genotyping was carried out by PCR using forward primer 5′-cga aggctacgtccaggagcgcaccat-3′, and reverse primer 5′-gcacggggccgtcgccgatgggggtgttctgc-3′, and by direct observation of EGFP-mediated fluorescence^36^. *Vangl2/EGFP* reporter mice were generated as reported^49^.

### EdU injection

For embryo staging, the morning after mating was defined as embryonic day (E) 0.5. Timed pregnant WT mice were injected intraperitoneally with EdU (100 μg/g mouse body weight) once at the indicated times (E11.5, E12.5, E13.5, E14.5, E16.5) and harvested on E18.5. *Atoh1/EGFP* reporter mice were injected three times with EdU at the indicated times (E11.5, E12.5, E13.5), and utricles were harvested 6 h after the initial injection.

### Immunocytochemistry and immunohistochemistry

The inner ear tissues were fixed in 4% paraformaldehyde (PFA; Electron Microscopy Sciences, Hatfield, PA, USA) for 30 min at room temperature. Utricles were processed for whole-mount preparations, or cryoprotected through a series of sucrose gradients, embedded in O.C.T. (Electron Microscopy Sciences), and sectioned to 8-μm thickness. Whole-mount immunostaining was carried out with 10% donkey serum (Millipore, Billerica, MA, USA) in 0.1% Triton X-100 in phosphate-buffered saline (0.1% PBST) for 1 h, followed by incubation with primary antibodies in 0.1% PBST overnight at 4 °C. On the second day, the tissues were rinsed 3–5 times in 0.1% PBST and incubated with the appropriate secondary antibodies in 0.1% PBST for 2 h at 37 °C under foil covers. The primary antibodies were against β-spectrin (1:100; BD Biosciences, San Jose, CA, USA), OCM (1:100; Santa Cruz Biotechnology, Santa Cruz, CA, USA), myosin VIIa (1:500; Proteus Biosciences, Ramona, CA, USA), p27^Kip1^ (1:100; Cell Signaling, Danvers, MA, USA), Pard6B (1:200; Santa Cruz Biotechnology, Santa Cruz, CA, USA), Gαi3 (1:400; Sigma, Sanit Louis, MO, USA), acetylated tubulin (1:200; Santa Cruz Biotechnology, Santa Cruz, CA, USA) and Prickle2 (1:500; gift from Professor Doris Wu). The secondary antibodies were conjugated with Cy5, FITC, or Rhodamine (1:1000; Jackson Laboratories). The tissues were then rinsed 3–5 times in 0.1% PBST and mounted for imaging. F-actin was stained with Alexa Fluor 488-conjugated (1:1000; Invitrogen) phalloidin for 30 min. For the staining of cryostat sections, the sections were permeabilised with 0.5% PBST and blocked with 10% normal goat serum. Subsequent procedures for whole-mount preparations were the same as those described above.

For detecting EdU incorporation, utricles were incubated with 1:1000 EdU (RiboBio, Guangzhou, China) in culture medium overnight at 37 °C before fixation with 4% PFA for 30 min. The procedures of EdU staining were carried out as described previously^50^. Immunofluorescence staining with other primary antibodies was performed immediately after EdU staining.

### Imaging and data analysis

Specimens were viewed using a Leica TCS SP8 laser scan confocal microscope with a 40 × or 63x objective lens (Leica, Wetzlar, Germany). At least three animals at each time point were used for immunofluorescence staining analysis. The quantification of Myo VIIa^+^, EdU^+^, or Myo VIIa^+^ and EdU^+^ cells was performed as described previously^51^. At least five samples at each stage under each condition were used for quantification. SPSS 22.0 software was used for statistical analysis. Data are presented as mean ± standard error. Two-tailed Student’s *t*-tests were performed for comparison between two groups, and p values ≤ 0.05 were considered statistically significant.

### SEM and quantification of hair cell planar polarity

Utricles were fixed with 2.5% glutaraldehyde (Electron Microscopy Sciences) in 0.1 M phosphate buffer (PB) at 4 °C overnight, post-fixed with 1% aqueous OsO_4_, dehydrated in a graded ethanol series, and dried by critical point drying with liquid CO_2_ (EM CPD300; Leica). Specimens were coated with 100 Å Au using an E-1045 sputter coater (Hitachi, Tokyo, Japan), and analysed with a NOVA NanoSEM 230 scanning electron microscope (FEI, Hillsboro, Oregon, USA) operated under a high vacuum at 5–10 kV at a working distance of 6–7 mm. The orientation of individual hair cells was measured using the ImageJ (NIH, USA) angle measurement tool. As outlined in Fig. 4A, stereocilia bundle polarity was measured in five analysis fields positioned in the posterior striola (PS), medial striola (MS), anterior striola (AS), medial extrastriola (MES), and lateral extrastriola (LES) separately. The detailed angle calculation method was performed as Deans described previously^29^. Vestibular hair cell orientation was assembled as a circular histogram using Matlab 2014a (Mathworks, USA) software.

## Additional Information

**How to cite this article:** Yang, X. *et al*. Establishment of planar cell polarity is coupled to regional cell cycle exit and cell differentiation in the mouse utricle. *Sci. Rep.* **7**, 43021; doi: 10.1038/srep43021 (2017).

**Publisher's note:** Springer Nature remains neutral with regard to jurisdictional claims in published maps and institutional affiliations.
